# Two developmental switch points for the wing polymorphisms in the pea aphid *Acyrthosiphon pisum*

**DOI:** 10.1186/2041-9139-4-30

**Published:** 2013-11-01

**Authors:** Kota Ogawa, Toru Miura

**Affiliations:** 1 Laboratory of Ecological Genetics, Graduate School of Environmental Science, Hokkaido University, Sapporo, Hokkaido 060-0810, Japan

**Keywords:** Wing polymorphism, Polyphenism, Developmental pathway, Developmental switch, Primordia formation, Wing bud, Flight muscle, Embryogenesis, Postembryonic development

## Abstract

**Background:**

In many insect taxa, wing polymorphism is known to be a consequence of tradeoffs between flight and other life-history traits. The pea aphid *Acyrthosiphon pisum* exhibits various morphs with or without wings associated with their complex life cycle including wing polyphenism in viviparous females, genetic wing polymorphism in males, and a monomorphic wingless phenotype in oviparous females and fundatrices. While wing differentiation has been investigated in some detail in viviparous females and males, these processes have not yet been elucidated in monomorphic morphs. The ontological development of the flight apparatus, including wings and flight muscles, was therefore carefully examined in oviparous females and fundatrices and compared with other morphs.

**Results:**

The extensive histological examinations showed that flight-apparatus primordia were not at all produced throughout their postembryonic development in oviparous females and fundatrices, suggesting that during the embryonic stages the primordia are degenerated or not developed. In contrast, in viviparous females and males, the differentiation points to winged or wingless morphs occurred at the early postembryonic instars (first or second instar).

**Conclusions:**

Based on the above observations together with previous studies, we propose that there are two developmental switch points (embryonic and postembryonic) for the flight-apparatus development in *A. pisum*. Since there are multiple developmental trajectories for four wingless phenotypes (wingless viviparous females, oviparous females, fandatrices, wingless males), it is suggested that the developmental pathways leading to various morphs were evolutionarily acquired independently under selective pressures specific to each morph. Especially in viviparous females, the delay of determination is thought to contribute to the condition-dependent expressions of alternative phenotypes, that is, phenotypic plasticity.

## Background

The ability of insects to fly, which is considered to have arisen only once in the insect class, has contributed enormously to their diversity and evolutionary success [1,2]. However, despite enabling insects to seek out new habitats, mates, and food resources, flight also incurs considerable costs for insects [1,3]. Consequently, numerous insect species have secondarily lost the ability to fly in favor of allocating energy toward traits such as fecundity, longevity, and weapons for intra- and interspecific competition [1,3,4]. In other words, as a result of tradeoffs between flying ability and other traits, wing polymorphisms and/or flightless phenotypes (c.f. brachypterous or apterous/wingless) have evolved in numerous insect taxa [5,6]. Some species express both winged and wingless phenotypes based on genetic and/or environmental factors, while other species express only wingless phenotypes that are secondarily derived from winged phenotypes [3,5,6].

In certain aphid lineages, genetic wing polymorphisms and environmental polyphenisms are both observed, sometimes even within a single species [7,8]. Although most aphid species exhibit a variety of phenotypes during their life cycle, wing polymorphisms, including wing polyphenism, are the most prevalent of these phenotypic changes [9]. In the instances of genetic wing polymorphism and environmentally-induced wing polyphenism, winged morphs have well-developed thoraces with fore- and hindwings, whereas wingless morphs have thinner thoraces and lack wings entirely [10,11].

Given that ancestral aphids are thought to have had winged adults, wingless morphs are considered to represent the derived condition [7,9,12,13]. Since the acquisition of wingless morphs appears to have contributed to the evolutionary success of aphids [7,8,13], the evolutionary transition and the acquisition of developmental mechanisms for producing wingless phenotypes are fascinating study foci.

**Figure 1:**
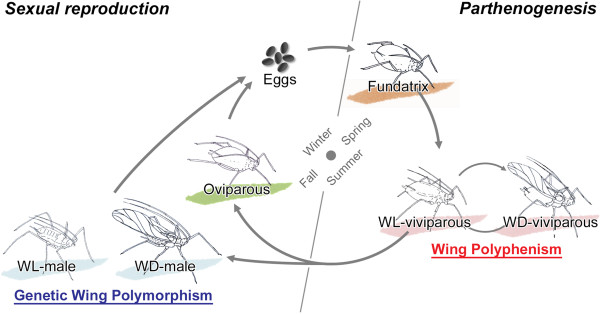
**Schematic diagram of annual life cycle in *A. pisum*.** This species reproduces by thelytokous parthenogenesis in spring and summer under long day length and high temperatures. Males and oviparous females only appear in late autumn and produce fertilized eggs for overwintering. Viviparous females exhibit wing polyphenism, and males exhibit genetic wing polymorphism. Fundatrices and oviparous females are monomorphic and wingless. WD: winged, WL: wingless.

The pea aphid *Acyrthosiphon pisum* (Harris 1776) exhibits a variety of wing polymorphisms associated with their complex life cycle [8]. As in other aphid species, *A. pisum* employs thelytokous and viviparous reproduction from spring to autumn. In response to a decrease in day length in late autumn, *A. pisum* produces males and oviparous females which then mate and lay overwintering eggs [14,15]. In the following spring, fundatrices, also referred to as ‘stem mothers’, hatch from the overwintering eggs, and then they parthenogenetically reproduce female progenies (Figure 1).

In *A. pisum*, several regulatory mechanisms are known to be involved in the wing polymorphisms/polyphenisms associated with the different reproductive modes observed in the annual life cycle of the aphid [8]. For example, in viviparous female generations, which exhibit wing polyphenism, unfavorable environmental conditions, such as high population densities or the presence of predators, can induce expression of the winged phenotype [13,16,17]. On the other hand, wing polymorphism in males has a genetic basis and the *aphicarus* (*api*) locus on the X-chromosome determines wing type (winged or wingless) [18-22], while oviparous females and fundatrices exist exclusively as monomorphic wingless phenotypes [8,9].

**Figure 2:**
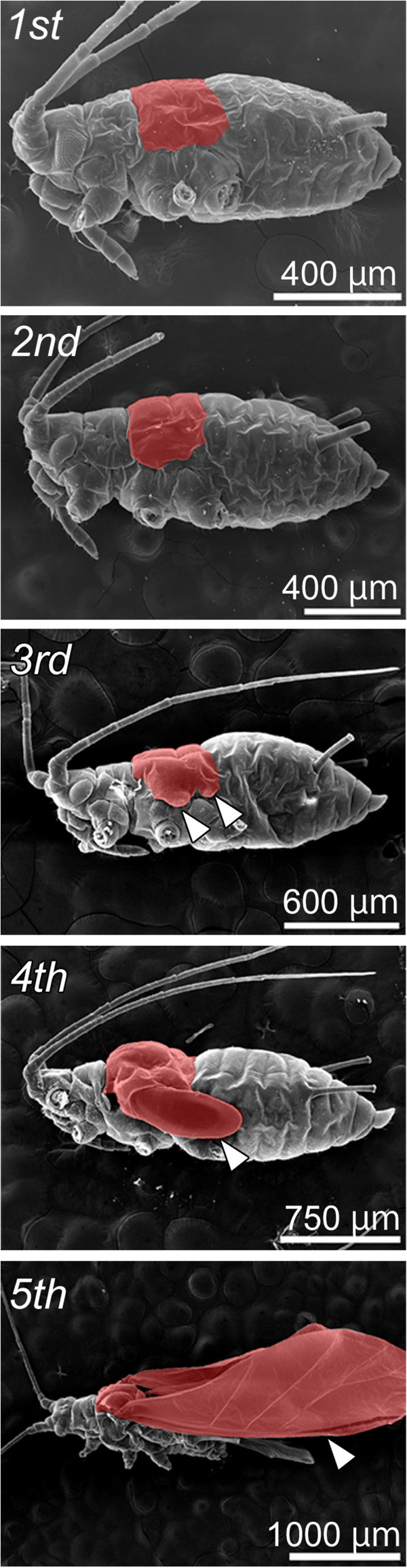
**External morphology in winged males of *A. pisum*.** Ordinal numbers indicate the nymphal stadia (instars). Meso- and metathoraces where wings are produced are indicated in red. Arrowheads indicate wings and wing primordia (third to fifth instars). Although no obvious external structures (bulges) are observed in first and second instar nymphs, they possess wing primordia underneath the cuticle (see Results for detail). No apparent differences of wing developmental process are found between the winged males and viviparous females. See the Additional file 1 for the methods of SEM observation.

**Figure 3:**
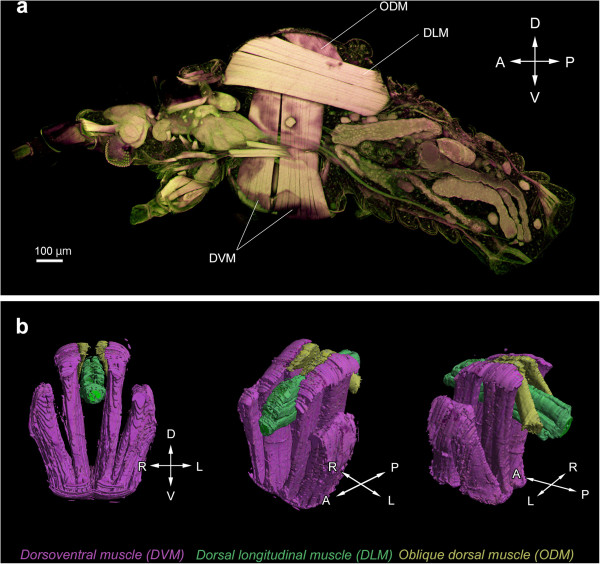
**Structure of the indirect flight-muscles in *A. pisum*. (a)** Conforcal laser scanning microscope (CLSM) image of the sagittal plane of winged male. **(b)** 3D reconstruntion images of the flight-muscles in the winged male. The indirect flight-muscles consist of three musculature components, that is, dorsoventral muscle, dorsal longitudinal muscle, and oblique dorsal muscle. No apparent differences of the muscles structures are found between the winged males and viviparous females. See the Additional file 1 for the methods of CLSM observation and 3D reconstruction. A: anterior, D; dorsal, DLM: dorsal longitudinal muscle, DVM: dorsoventral muscle, L: left, ODM: oblique dorsal muscle, P: posterior, R: right, V: ventral.

The winged morphs in males and viviparous females of *A. pisum* possess the functional flight apparatus that share the homologous structures (that is, wings and flight muscles) [10,11,23]; the wings are developed sufficiently to gain aerodynamic lift (Figure 2, see the Additional file 1 for the observation method), and indirect flight muscles which consist of dorsoventral muscles (DVM), dorsal longitudinal muscles (DLM), and oblique dorsal muscles (ODM), are well-developed to flap their wings (Figure 3, see the Additional file 1 for the observation method). On the other hand, developmental differences of flight muscles are seen between wingless morphs in males and viviparous females [11]. Previous studies on female wing polyphenism reported that the first-instar nymphs of wingless viviparous females possess wing and flight-muscle primordia, which then degenerate during postembryonic development [10,23]. However, in examples of male wing polymorphism, the flight muscles of wingless morphs were observed to be developed and differentiated, but probably not functional [11]. These findings suggest that, although both male and female wingless forms have similar developmental patterns in terms of external morphology, the regulation of flight apparatus development differs between the two wingless forms.

Similarly, although the flight-apparatus development/degeneration patterns in oviparous females and fundatrices also appear to differ from the patterns observed in other wingless morphs to date, little is known about the developmental processes involved in flight apparatus development in monomorphic wingless morphs. Since oviparous females and fundatrices are wingless in most aphid species, analyses of these two wingless morphs may provide insights into the evolution of wingless phenotypes in aphids [8,13].

Therefore, in this study, detailed histological observations were conducted to compare the patterns of flight apparatus development during embryogenesis and postembryonic development in all *A. pisum* morphs, and to elucidate the various evolutionary processes leading to flightlessness in aphids. We also examined whether the flight-muscle breakdown reported in winged viviparous females [23] also occurs in adult males in order to provide further insight into the wing polymorphisms in *A. pisum*.

## Methods

### Insects

Three established aphid strains of *A. pisum*, ApL, 08Ap1, and 06D, that were originally collected in Sapporo, Japan, were used in this study (Table 1). All three strains exhibit heterogony (that is, they have both parthenogenetic and sexual generations) and the male wing phenotypes of these strains differ (see below for details). Stock aphid populations were maintained for several generations under long-day conditions (16L/8D, 20°C) in test tubes (diameter: 2.5 cm, height: 10 cm) containing vetch seedlings (*Vicia faba*) grown on wet vermiculite [24].

**Table 1:** Induction methods and focal strains

**Morph**	**Strain**	**Induction method**	**References**
Wingless male^a^	08Ap1, 06D	Rearing mother aphids (viviparous) under short-day conditions	[8,11,25]
Winged male^a^	ApL, 06D	Rearing mother aphids (viviparous) under short-day conditions	[8,11,25]
Wingless viviparous female^b^	ApL	Rearing mother aphids (viviparous) under low-density conditions	[8-10]
Winged viviparous female^b^	ApL	Rearing mother aphids (viviparous) under high-density conditions	[8-10]
Oviparous female (wingless)^c^	ApL	Rearing mother aphids (viviparous) under short-day conditions	[9,25]
Fundatrix (wingless)^c^	ApL × ApL	By mating male and oviparous female	[9,13,26]

See Methods for detail of induction method.

^a^Genetic wing polymorphism.

^b^Wing polyphenism.

^c^Monomorphic wingless.

In *A. pisum*, male wing polymorphism is determined by a single gene locus, *aphicarus* (*api*), that is located on the X-chromosome [18-21]. Therefore, depending on the maternal genotype, all homozygous *api* male progeny are either winged or wingless, and heterozygous males would be both winged (50%) and wingless (50%) [19,21,22]. ApL and 08Ap1 strains exclusively produce winged and wingless males, respectively, while the 06D strain produces both winged and wingless males in a 1:1 ratio. The wing type of male nymphs could be discerned based on their strain and wing-bud development [11].

### Induction of sexual generation and fundatrices

Since the switch from asexual to sexual reproduction in *A. pisum* is triggered by short-day length and low temperature [15], first-instar nymphs produced by wingless aphids were individually reared on seedlings at 15°C under 8L/16D. Under these conditions, males and oviparous females of the three focal strains were produced across several generations as described previously, although the rate of morph production differed among strains [11,25]. For fundatrix induction, one male and three oviparous females were placed together for approximately 10 days in a plastic case containing a vetch seedling. Fertilized eggs deposited onto the seedlings were then transferred to plastic cases containing moist filter paper and maintained at 15°C for 2 weeks and then 4°C for 50 days. Eggs hatched approximately 10 days after transferring to 15°C [26].

### Induction of winged and wingless viviparous females

In *A. pisum*, physical contact at high densities is known to be the key stimulus inducing the production of winged viviparous females [17]. Specifically, mother aphids produce winged progeny under high-density conditions and wingless progeny under low-density conditions [17]. In order to obtain winged and wingless female morphs for the wing polyphenism experiments, we manipulated density conditions to induce both wing types as described previously [10]. For the high-density condition, more than 30 wingless adults were reared on a single 3-cm high vetch seedling and the resulting winged progeny were collected. Conversely, for the low-density condition, only one wingless adult was kept on a 3-cm high vetch seedling and the resulting wingless progeny were collected.

### Histological examination

To compare flight-apparatus development among morphs, paraffin sections were prepared as described previously [10]. Briefly, specimens were fixed in FAA fixative (formalin: alcohol: acetic acid = 6:16:1), dehydrated in increasing concentrations of ethanol, and transferred to xylene before being embedded in paraffin. Serial sections (5-μm thick) were processed routinely and stained with hematoxylin and eosin. Tissues were observed under a light microscope (BX-51, Olympus, Tokyo), and images were captured using a CCD camera (DP-72, Olympus) and software (DP2-BSW, Olympus).

### Comparison of wing primordia from oviparous and viviparous females during embryogenesis

In viviparous females, late-stage embryos already have wing and flight-muscle primordia [23]. To determine whether the flight-apparatus primordia are formed in embryos that are destined to become oviparous females, aphids were fixed and paraffin sections were prepared. The morphs of embryos were identified using strain-specific reproductive patterns and assessments were confirmed by examining the ovarian structure at the same time the flight apparatus was examined. Specifically, embryos destined to become oviparous females were obtained from viviparous adult aphids that had been reared under short-day and low-temperature conditions (8L/16D, 15°C) for two generations [25]. Embryos destined to become wingless viviparous females were obtained from adult aphids reared under long-day (16L/8D, 20°C) and low-density conditions. The degree of cell proliferation in thoracic epithelia (where wing primordia form) was compared between corresponding embryonic stages using the embryonic stages of Miura et al. [27].

### Comparison of flight-muscle breakdown in winged viviparous females and males

Although flight-muscle breakdown has been reported previously in viviparous winged females [23,28], flight-muscle breakdown is not known to occur in males. To investigate whether flight-muscle breakdown also occurs in males, paraffin sections of the flight apparatus from younger adult males were prepared immediately after the imaginal molt. The older males were sampled 10 days after the imaginal molt and were confirmed to have mated with oviparous females.

## Results

### Flight-apparatus development during postembryonic development 1: males and viviparous females

**Figure 4:**
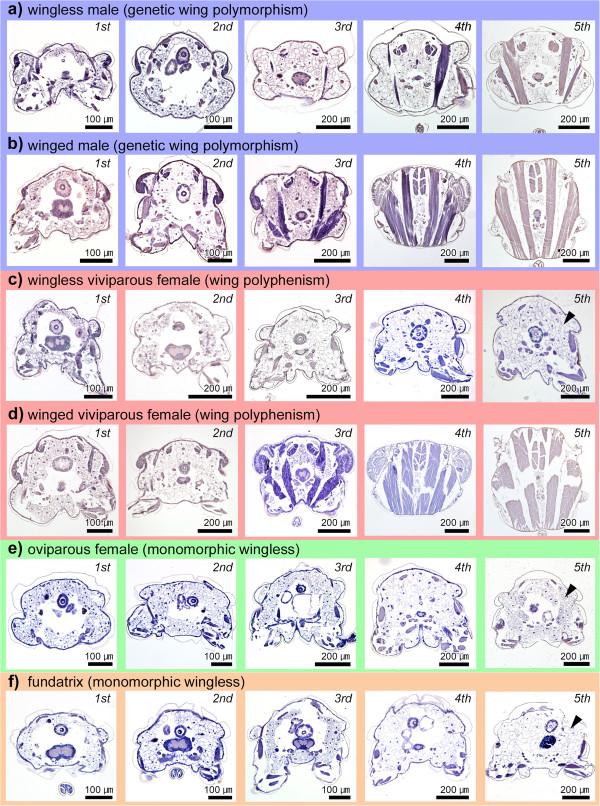
**Histological comparisons of postembryonic flight-apparatus development among all *A. pisum *morphs. (a)** Wingless male; **(b)** winged male; **(c)** viviparous wingless female; **(d)** viviparous winged female; **(e)** oviparous female; **(f)** fundatrix. Transverse planes of second to third thoracic segment are shown. Although wingless males do not possess wings, they possess flight muscles. In winged adults, wings were removed before the sample preparations. Ordinal numbers indicate the nymphal stadia (instars). Arrowheads indicate fat cells.

**Figure 5:**
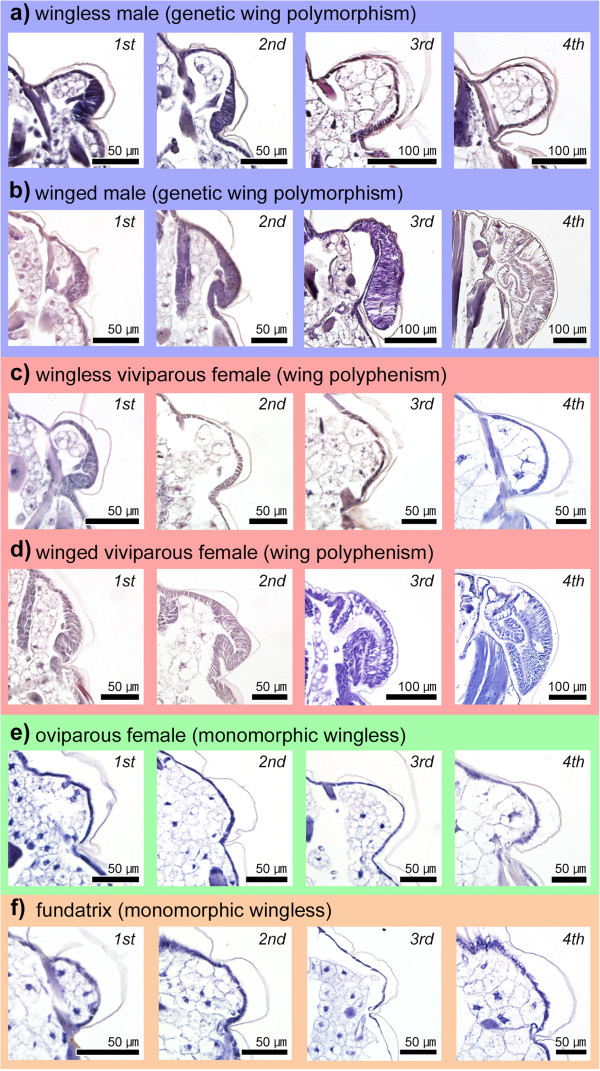
**Histological comparisons of wing primordial development/degeneration among morphs. (a)** Wingless male; **(b)** winged male; **(c)** wingless viviparous female; **(d)** winged viviparous female; **(e)** oviparous female; **(f)** fundatrix. Transverse planes of second to third thoracic segment are shown. Wing primordia in wingless viviparous females and wingless males degenerate during postembryonic development. Primordia are not observed in fundatrices and oviparous females. Ordinal numbers indicate the nymphal stadia (instars).

To compare the processes associated with flight-apparatus development (that is, wing buds and flight muscles) among all morphs, the internal structures of the thoracic segments were observed throughout the postembryonic development. In both winged and wingless males, all of the first instar nymphs possessed wing primordia (Figures 4a, b, 5a, b). In second instar nymphs, wing primordia had developed and become thickened in winged males (Figure 5b), but primordial growth had stopped in wingless males (Figure 5a). In third instar nymphs, wing primordia (wing buds) became markedly larger and thicker in winged males (Figure 5b), but they disappeared entirely in wingless males in which only flattened epithelia were observed (Figure 5a). After molting into the fourth instar, the wing primordia (wing pads) of winged males were shaped like a sheath, within which the wing epithelia were folded in a complicated manner (Figure 5b). Regarding flight muscles, both winged and wingless males possess flight-muscle primordia (for example, myoblasts), which developed and differentiated even in wingless males (Figure 4a, b). These observations of third and fourth instar males closely corroborated those of our previous study [11]. As previously reported [11], the flight muscles of wingless males consist of three components, that is, dorsoventral muscle, dorsal longitudinal muscle, and oblique dorsal muscle, as seen in winged males, despite the degree of flight muscle development differs. Thus, in wingless males, flight-muscle development is just suppressed and the muscle breakdown during the postembryonic development does not occur, unlike viviparous females (see below for details).

In addition, the postembryonic development of the flight apparatus in winged males was similar to that observed in winged viviparous females (Figures 4c, d, 5c, d) [10,23]. However, despite possessing wing and flight-muscle primordia, the developmental processes and timing of flight-apparatus development/degeneration in wingless viviparous females and males differed markedly (Figure 4a, c). In wingless viviparous females, wing primordia disappeared by the second instar (Figure 5c) and in wingless males, by the third instar (Figure 5a). Flight-muscle primordia in wingless viviparous females degenerated by the second instar (Figure 4c) [10,23], while wingless males possessed flight muscles throughout their lives (Figure 4a).

### Flight-apparatus development during postembryonic development 2: oviparous females and fundatrices

In oviparous females and fundatrices, no evidence of immature or vestigial wing tissues (for example, thickened epithelia) or flight-muscle primordia (for example, myoblasts) was observed in the thorax throughout nymphal development, that is, from the first to the fourth instars (Figures 4e, f, 5e, f). Thus, adult oviparous females and fundatrices were both considered to lack the flight apparatus; instead, both life stages possessed fat cells in the thoracic region (Figure 4e, f) like wingless viviparous females (Figure 4d). Further, the internal anatomy of these adults appeared similar to that of wingless viviparous females (Figure 4c).

### Comparison of wing primordia of oviparous and viviparous females during embryogenesis

**Figure 6:**
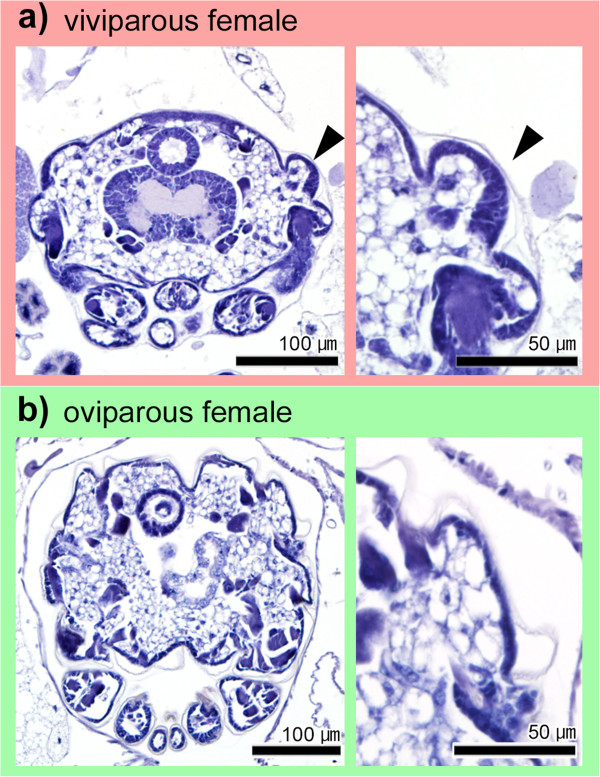
**Comparison of wing primordia of a viviparous female (a) and an oviparous female (b) during embryogenesis.** Transverse planes of thoracic segments are shown. Left and right panels show whole and magnified images, respectively. Although wing primordia are not observed in oviparous embryos **(b)**, viviparous embryos possessed primordia after stage 18 **(a)**. Arrowheads indicate wing buds.

Based on the observation that flight-apparatus primordia were absent in the first instar of oviparous females and fundatrices, two possible scenarios for flight apparatus development in monomorphic wingless morphs can be proposed; no primordia formed, or the primordia formed and then were subsequently degraded during embryogenesis. To evaluate the relative likelihood of these two scenarios, embryogenesis in oviparous and viviparous females was observed. In viviparous females, although wing primordia were not observed at embryonic stages 16 to 17 (that is, from the end of katatrepsis to immediately before eye differentiation), the primordia were detected at stages 18 to 20 (that is, from the initiation of eye differentiation to birth) (Figure 6a). On the other hand, no wing primordia were detected in oviparous female embryos at stages 18 to 20 (Figure 6b). The flight-muscle primordia (myoblast) were not clearly identified in both embryos because the embryos contained a number of myoblasts. Since the embryos of fundatrices, unlike oviparous and viviparous females, enter diapause at an early stage in overwintering eggs [26], comparisons between fundatrices and other females were realistically difficult.

### Comparison of flight-muscle breakdown in winged viviparous female and males

**Figure 7:**
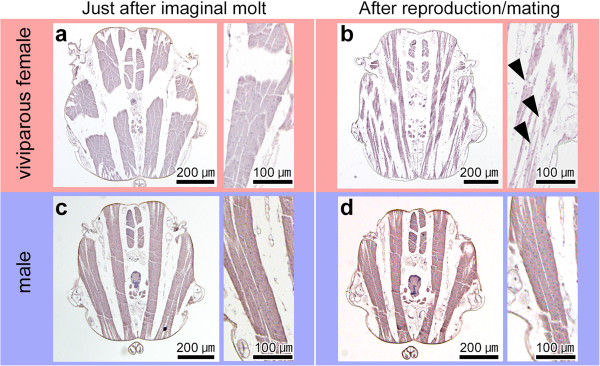
**Comparison of flight-muscle breakdown between viviparous females (a, b) and males (c, d).** Transverse planes of thoracic segments are shown. Left and right panels show whole and magnified images, respectively. Although the flight-muscles degenerated immediately before larviposition in viviparous females **(b)**, no degeneration was observed in males even after mating **(d)**. Arrowheads indicate degenerating muscles.

To investigate whether the flight-muscle breakdown that occurs in winged viviparous females (Figure 7a, b) [23] also occurs in males, we compared the flight muscles of younger and older adult males (Figure 7c, d). Even in older males, which had mated more than 10 days after the imaginal molt, flight muscles with functional fibrous architecture were observed (Figure 7d). In addition, no differences were observed in the flight muscles and architecture between older and younger adult males (just after imaginal molt) (Figure 7c), and no flight-muscle breakdown was observed in the older wingless males (data not shown).

## Discussion

**Figure 8:**
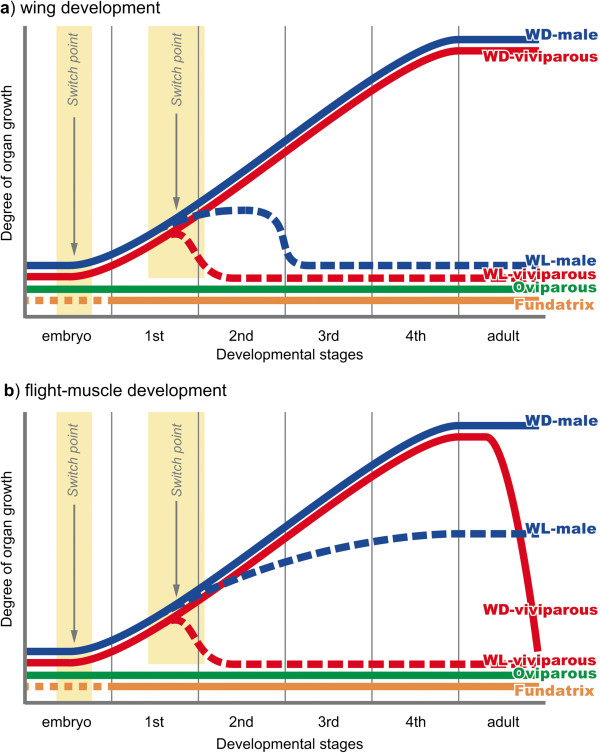
**Schematic diagrams of wing (a) and flight muscle (b) development.** Development times differ slightly between wings and flight muscles. No flight-apparatus primordia are observed over the course of a lifetime in both fundatrix and oviparous females, which are wingless. However, in wingless males and wingless viviparous females, wing primordia degenerate during early postembryonic development. Even though flight muscles develop and differentiate in wingless males, the flight-muscle primordia in wingless viviparous females also degenerate. Furthermore, in winged viviparous females, flight-muscle breakdown and energy reallocation both occur before larviposition starts. WD: winged, WL: wingless.

In this study, detailed histological observations revealed morph-specific differences in developmental patterns of flight apparatus in all morphs of *A. pisum*, with differences being most apparent among wingless morphs (Figure 8). As reported in previous studies [10,23], flight-apparatus primordia formed in both wingless and winged viviparous female aphids, although the primordia disappeared during the early nymphal instars (Figures 4c, 5c, 6a).

However, to the best of our knowledge, this is the first study to demonstrate that flight-apparatus primordia did not form during embryogenesis or postembryonic development in oviparous females (Figures 4e, 5e, 6b), or during postembryonic development in fundatrices (Figure 4f). Taken together, these findings showed that wing primordia did not develop at all in any of the developmental stages in these morphs. Since we were unable to observe embryogenesis in fundatrices, it is possible that they possess wing primordia during embryonic development, but they completely degenerate before hatching from their overwintering eggs. In most aphid species, oviparous females mate and reproduce on their natal host plants in autumn, and fundatrices resume their development in early spring when their food sources (plant hosts) are restricted and it is difficult to disperse to new habitat [13,29]. Consequently, since producing winged morphs of these two phenotypes would be non-adaptive, it is possible that the developmental patterns leading to the production of wingless morphs may have evolved multiple times as the development of flight-apparatus primordia would be unnecessary.

In the case of wingless males, the wing primordia are once formed but degenerated after the second instar (Figure 5a). The timing of wing degeneration differs from that observed in viviparous wingless females, which occurs at an earlier stage, that is, in the late first instar (Figure 5c). In addition, unlike wingless females, wingless males possess flight muscles (Figure 4) [11], implying that flight muscle development in wingless males is merely suppressed and that muscle breakdown does not occur. Indeed, it is likely that flight muscle development is limited by the relatively short total nymphal period of wingless males [11]. Similarly, no flight-muscle breakdown occurs in winged males after mating, while flight-muscle breakdown has been reported in winged females after the dispersal flight (Figure 7) [23,30]. This is because, unlike winged viviparous females, shunting the energy derived from muscle degeneration to reproductive organs would not be necessary in winged males [10,11,23,30]. Based on our extensive comparisons among all morphs in *A. pisum*, it is suggested that there are two developmental switch points for the divergences in wing and in flight-muscle development: one in the embryo and one in the early nymphal instars, that is, first to second instar stages (Figure 8).

**Figure 9:**
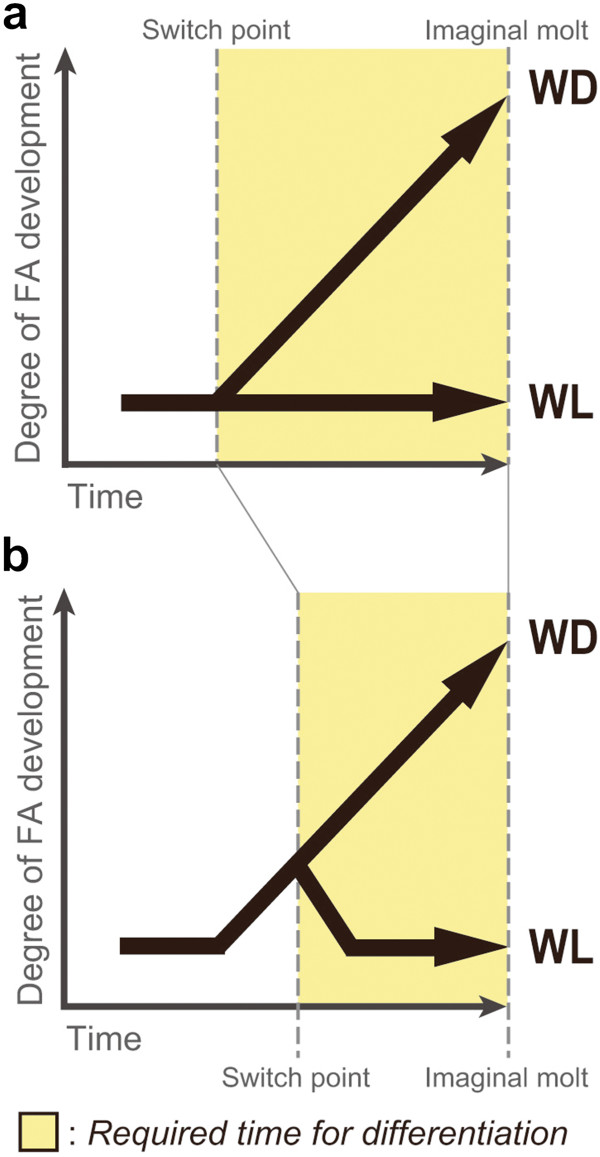
**Schematic diagrams showing the hypothesized relationships between the developmental time in both winged and wingless aphids and the degree of flight-apparatus development. (a)** The forked pathway in which wing primordia did not develop at all in any of the developmental stages in wigless morphs; **(b)** the pathway in which wing primordia once organize and breakdown in wigless morphs. In polyphenic development, the pathway including primordia formation and subsequent context-dependent degeneration **(b)** may be maintained rather than the pathway without unavailing primordia **(a)** due to allowing rapid response to environmental cues. FA: flight apparatus, WD: winged, WL: wingless.

The timing of differentiation into distinctive developmental pathways generally depends on certain selective pressures and/or developmental constraints [31,32]. If selective pressure favors the development of wingless phenotypes, the onset of differentiation would occur earlier in order to accomplish wing degeneration as quickly as possible, and in so doing, save on the costs associated for wing formation (Figure 9a). Thus, in the case of *A. pisum*, ‘complete’ winglessness is favored for oviparous females and fundatrices which do not form primordia at all.

Conversely, if selection favors a certain amount of flexibility to adapt to unstable environments regarding the development of phenotypes with both wing types, the switch point can be organized later in development (Figure 9b). In other words, the pathway including primordia formation and context-dependent degeneration may contribute to rapid response to environmental cues rather than vestigial non-functional tissues (Figure 9). Therefore, in the focal species, retaining the potential to two developmental pathways is favored for wingless viviparous females possessing flight-apparatus primordia, allowing them to have the ability to respond to multiple environmental cues, such as density conditions [17]. Therefore, to sustain the potential to produce progeny with both wing types (totipotency), the developmental pattern in which primordia are formed and then subsequently degenerated may have been necessary to produce wingless morphs. In the case of the male genetic wing polymorphism, since the resources required for reproduction are not as limiting as they are in females, wing degeneration is not as well developed as it is in wingless viviparous females. In addition, as wingless males are considered to have evolved later than other wingless phenotypes [8,12], the pathway for degenerating primordia that have already formed may not have evolved.

In terms of the physiological and molecular bases for the wing polymorphism/polyphenism, a number of previous studies suggest that the suppression of wing development require for high juvenile hormone titer (reviewed in [7,8]). Furthermore, it is known that the expression level of *apterous1*, a homolog of *apterous* gene involved in the wing morphogenesis, differs between winged and wingless viviparous females in the first to the second nymphal instars [33]. However, the molecular and physiological knowledge on the wing polyphenism/polymorphism is concentrated in viviparous females, and comparisons with other morphs have been carried out only during the late instars. Therefore, to fully understand the developmental regulations of wing and flight muscles in *A. pisum*, further molecular and physiological analyses encompassing all morphs and developmental stages should be required.

In this study, histological analyses were conducted to compare the developmental patterns in flight apparatus development during embryogenesis and postembryonic development in all morphs of *A. pisum*. Looking across all aphid taxa, some aphid species have monomorphic winged morphs despite exhibiting dispersal polymorphisms with respect to behavior, while others have brachypterous morphs [9,12,13,34,35]. Thus, considering the evolutionary processes leading to flightlessness in aphids, comparative analyses among these aphid species will have profound implications for our understanding of the evolution of wing polymorphisms in insects.

## Conclusions

This study revealed that, by extensive histological observations, morph-specific differences in flight apparatus development patterns in all morphs of *A. pisum*, with differences being most apparent among wingless morphs. The results showed that, unlike viviparous females and males, no flight-apparatus primordia were produced in monomorphic wingless morphs. Based on these observations, we propose that the flight-apparatus development in *A. pisum* is regulated by two developmental switch points in the embryo and in the early nymphal instars. Since there are multiple developmental trajectories for different phenotypes, it is suggested that the developmental pathways leading to various morphs were evolutionarily acquired independently under selective pressures specific to each morph.

## Abbreviations

A: Anterior; CLSM: Conforcal laser scanning microscope; D: Dorsal; DLM: Dorsal longitudinal muscle; DVM: Dorsoventral muscle; FA: Flight apparatus; FAA: Formal acetic alcohol; L: Left; O: Oviparous female; ODM: Oblique dorsal muscle; P: Posterior; R: Right; WD: Winged; WL: Wingless; V: Ventral.

## Competing interests

The authors declare that they have no competing interests.

## Authors’ contributions

KO designed the study, performed the experiments and drafted the paper. TM participated in the design of the study and revised the manuscript critically. Both authors read and approved the final manuscript.

## Supplementary Material

Additional file 1 Supplemental methods. Click here for file
