# Supplementary material for: Two developmental switch points for the wing polymorphisms in the pea aphid Acyrthosiphon pisum
Source: EvoDevo. 2013 Nov 1;4:30. doi: 10.1186/2041-9139-4-30 (PMC3843552; doi:10.1186/2041-9139-4-30)
Supplement: Additional file 1 — Supplemental methods. [file 2041-9139-4-30-S1.docx]

**Supplemental methods**

**Scanning electron microscopy (Fig. 2)**

Scanning electron microscopy (SEM) was used to describe the developmental process of wings (wing buds) in winged aphids. Aphid samples of all instars were fixed in FAA solution (formalin:ethanol:acetic acid, 6:16:1) and transferred into increasing concentrations of ethanol followed by t-butanol. After that, they were freeze-dried using an ES-2030 freeze dryer (Hitachi, Tokyo) and coated with gold ions with an E-1010 ion sputter (Hitachi, Tokyo). The detailed morphological characteristics of thoracic structures were observed with a scanning electron microscope (JSM-5510LV; JEOL, Tokyo).

**Confocal laser scanning microscopy (Fig. 3a)**

Confocal laser scanning microscopy (CLSM) was used to image anatomical structures of the flight-muscles. Specimens were ﬁxed in FAA ﬁxative (formalin: alcohol: acetic acid = 6:16:1). Fixed specimens were sliced by ophthalmic surgical knife (Micro Feather Blade, Feather, Osaka) and transparentized by FocusClear^TM^ (CelExplorer Labs Co., Taiwan). Observations were carried out using a Carl Zeiss LSM510 META laser-scanning confocal microscope equipped with a laser unit: Argon/2 and DPSS 561-10, HeNe633. We collected the autofluorescence excited by 488nm, 561nm and 633 nm wavelengths with three channels using 10× Plan Achromat objectives. Stacking images were merged into a single image and adjusted contrast by Adobe Photoshop CS6.

**3D reconstruction of the flight muscle (Fig. 3b)**

3D images of the flight muscles were reconstructed from histological sections using a free software package Delta Viewer (version 2.1.1, <http://delta.math.sci.osaka-u.ac.jp/DeltaViewer/>).
